# Molecular Dissection of the Basal Clades in the Human Y Chromosome Phylogenetic Tree

**DOI:** 10.1371/journal.pone.0049170

**Published:** 2012-11-07

**Authors:** Rosaria Scozzari, Andrea Massaia, Eugenia D’Atanasio, Natalie M. Myres, Ugo A. Perego, Beniamino Trombetta, Fulvio Cruciani

**Affiliations:** 1 Dipartimento di Biologia e Biotecnologie “Charles Darwin”, Sapienza Università di Roma, Rome, Italy; 2 Sorenson Molecular Genealogy Foundation, Salt Lake City, Utah, United States of America; 3 AncestryDNA, Provo, Utah, United States of America; 4 Dipartimento di Biologia e Biotecnologie “Lazzaro Spallanzani”, Università di Pavia, Pavia, Italy; University of Florence, Italy

## Abstract

One hundred and forty-six previously detected mutations were more precisely positioned in the human Y chromosome phylogeny by the analysis of 51 representative Y chromosome haplogroups and the use of 59 mutations from literature. Twenty-two new mutations were also described and incorporated in the revised phylogeny. This analysis made it possible to identify new haplogroups and to resolve a deep trifurcation within haplogroup B2. Our data provide a highly resolved branching in the African-specific portion of the Y tree and support the hypothesis of an origin in the north-western quadrant of the African continent for the human MSY diversity.

## Introduction

The male specific portion of the Y chromosome (MSY) is the largest haplotypic block in the human genome. Due to the lack of allelic recombination, the history of mutational accumulation in the MSY can be described in a unique evolutionary haplogroup tree. The MSY tree has been largely used for the study of the human evolutionary history [1], [2], and is currently becoming an increasingly important resource in forensic [3], [4] and sex-chromosome evolution studies [5]–[13].

The number of phylogenetically characterized Y-specific markers has substantially increased in recent years. In the MSY phylogeny reported by Karafet et al. [14], 80 mutations belong to the deepest portion of the tree (haplogroups A1a, A1b, A2, A3 and B), defining a total of 34 distinct African-specific haplogroups/paragroups. An independent high resolution MSY phylogeny has been recently obtained from 2,870 Y-SNPs discovered (or re-discovered) in the course of a large whole-genome re-sequencing study, but the observed variable sites all belong to the recent “out of Africa” CT clade [15]. Recently, in a re-sequencing study of the Y chromosome, the root of the tree moved to a new position and several changes at the basal nodes of the phylogeny were introduced [16]. Interestingly, the estimated coalescence age and deep branching pattern of the revised MSY tree appear to be more similar to those of the mtDNA phylogeny [17], [18] than previously reported [1].

To increase the level of phylogenetic resolution in the African-specific portion of the MSY tree, we mapped 146 previously detected mutations [16] with respect to 59 mutations defining internal nodes in the Y chromosome phylogeny reported by Karafet et al. [14]. We also mapped eleven previously unreported mutations from our database, and nine new mutations that had been identified through the re-sequencing of two Y chromosomes belonging to the widespread African-specific haplogroup A3b. Finally, two additional mutations identified during the process of phylogenetic mapping were also included in the analysis. The new integrated MSY tree represents an improved resource for the study of human prehistory and genome evolution.

## Results

### Newly Described Mutations

Information regarding 22 previously undescribed mutations is given in Table 1. The first set of eleven mutations came from our database (mutations in the range V2-V249 in Table 1). Nine new mutations (mutations in the range of V262-V317 in Table 1) were discovered by sequencing two A3b subjects, while two additional mutations (V254 and V341) were identified during the process of phylogenetic mapping. Apart from one exception (V306, corresponding to the SNP rs113042298), none of these mutations is present in the current SNP database (dbSNP, build 135).

**Table 1:** Information on the mutations here reported and analyzed for the first time.

SNP	Y-Position^a^	Mutation	Forward Primer	Reverse Primer
V2	6778215	A to C	TTGCTGAGTGTACTGGGATCTT	AAGACACCCCAGTTGTCATTTC
V3	6778229	T to C	TTGCTGAGTGTACTGGGATCTT	AAGACACCCCAGTTGTCATTTC
V11	6892902	A to T	CTGGCCTTAGGAAATAGGTCAA	AGGGTCATTATCGTTGAGGAAG
V33	6894717	G to T	AACACCAGGATTTGTTTTGGAG	TTCAGCAACACTGTAGAATCAGG
V34	6894718	C to T	AACACCAGGATTTGTTTTGGAG	TTCAGCAACACTGTAGAATCAGG
V37	6818279	G to A	ATTATTTGAATGCAAGTGGGGA	TGAAACTGAATTAAAGGAAGGTGG
V73	16691696	A to G	TGAAGGAGATTTAATTGGGGTAGA	TTTCCATACCATGCCTTTCTTT
V87	17947454	A to T	CATCCCTTGGTCATCCCTC	CTAGACCCTGATTCATGTAAGCC
V147	6739492	G to A	GTTTTGTGGGTGAGAGAGGAAG	CCATTTCTACATGGAGGAAGTTTT
V248	7589991	C to T	GGCAGCCCTCAGGATATGTA	GATTCCACTAAACCCGACGA
V249^b^	25207704; 26841450;27120952	T/T/T toG/G/G	GGCCAAATCAGAGAAATGGA	CGCAGAACCTGAAATTGTGA
V254	6870497	G to A	AAATGCAGTGTTCCAGGGAGT	TTAAGTAGCTCCCGAGAAGTTAAAG
V262	6659209	C to G	CTACAACGCCCAGCTGATTT	CTGTCTTATGGCCACCCAAG
V265	6661164	A to G	CTCTAGCAATTAGGGCTTCAG	TGTTGCCTAGATGACAAGCA
V303	2798066 - 2798068	del TTT	GGCACCCTGTAGAACCCATA	GAAAAAGAGCAGGCATGGTG
V304	2796955 - 2796957	del GAA	GCTTTGGGGGAGTTAGGAAA	TACAGGGTGCCAGATGGACT
V305	2854573	T to C	ACCCCTGGGTTCAACTATCC	ATTACTCAGGGGTGGTGACG
V306	7594967	G to C	CCTTAAAGGGCTCGGAGAGA	CACTCGACATCGACCTCTCA
V313	7622390	G to A	GAGACTCAGGCAGGCATCAT	TCCAAAGGTTCACAGCTGATT
V314	7642949	A to T	AGGCCTCATCCAGACCTATG	CCACCATGCTGGCTTATTTT
V317	2908553	G to A	AAGATGCCAGCCTCGAGTTA	TTTTTGATCTGAGGCCCATC
V341	4840884	A to T	TTCAGCTATTGCCTTCTATGG	TTTAAGCTCGTGGGAAATGC

a Position according to the February 2009 human Y-chromosome reference sequence (GRCh37).

b PCR primers amplify three paralogous MSY regions.

### Phylogenetic Mapping

**Figure 1:**
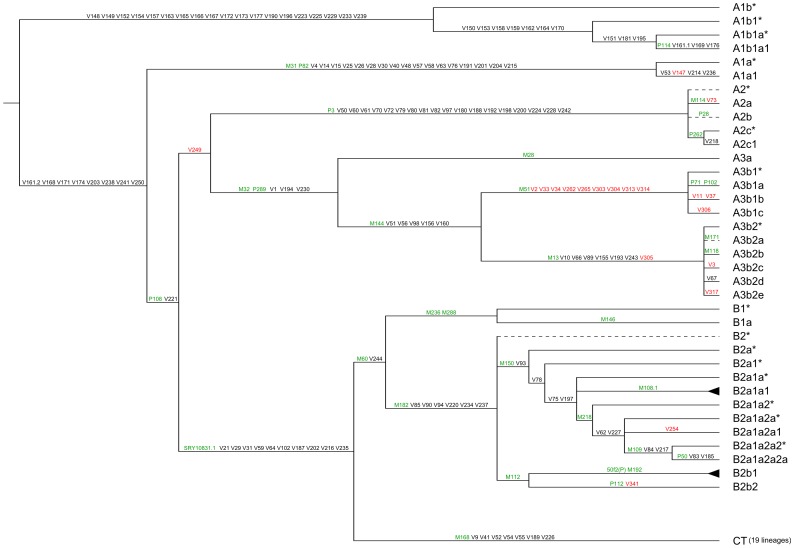
Revised topology of the deepest portion of the human MSY tree. The names of the mutations genotyped are indicated on the branches (green, mutations from the paper by Karafet et al. [14]; black, mutations from the paper by Cruciani et al. [16]; red, previously undescribed mutations, see text). For the sake of clarity, the internal structure of haplogroups B-M108.1 (2 branches) and B-50f2(P) (8 branches) is not shown (black triangles). The phylogenetic position of mutations mapping within haplogroup CT is shown in Figure S1. Dashed lines indicate putative branchings (no positive control available). The microsatellite intermediate allele DYS449.2, that was found to delineate new phylogenetic structure in human Y chromosome haplogroup tree [42], was not observed in 19 Y*(xBT) and 4 B chromosomes analyzed.

Most of the mutations here analyzed belong to the African portion of the MSY phylogeny, which is comprised of haplogroups A1b, A1a, A2, A3 and B [16]. Through phylogenetic mapping it was possible to identify 15 new African haplogroups and to resolve one basal trifurcation (Figure 1). A new deep branch within the “out of Africa” haplogroup C was also identified (Figure S1).

#### Haplogroup A1b

The P114 mutation, which defines haplogroup A1b according to Karafet et al. [14], had been detected in central-western Africa at very low frequencies (in total, three chromosomes from Cameroon) [16], [19]. Thirty-two additional biallelic mutations have been recently discovered for haplogroup A1b, which now appears to be one of the two deepest-rooting branches of the MSY tree [16]. Here, these mutations were analyzed in three Y chromosomes that were ancestral at both P114 and the markers that, according to [16], define macro-haplogroup A1a-T (samples 1–3 in Table S1) and in one Y chromosome derived at P114 (sample 4 in Table S1). The four chromosomes turned out to carry the derived allele at 19 of the 32 markers analyzed, resulting in one terminal haplogroup, defined by P114 and three other mutations, and three new paragroups (Figure 1). We identified a third allele (A) for the V161 polymorphism, which had been previously reported as a biallelic G to C tranversion on the A1b branch [16]. The presence of nucleotide A at the orthologous MSY position in the chimp reference sequence (October 2010 chimp assembly, UCSC Genome Browser), along with the structure of the human MSY tree as shown in Figure 1, suggest that this triallelic polymorphism may have originated from two independent mutations, from A to C (V161.1) within the A1b branch, and from A to G (V161.2) at the root of macro-haplogroup A1a-T.

#### Haplogroup A1a

Haplogroup A1a is present at relatively low frequencies in western and northern Africa [20]–[25]. In the phylogeny by Karafet et al. [14], A1a is defined by mutations M31 and P82. Twenty additional mutations have been reported for this branch [16]. These mutations, along with M31, P82 and a mutation identified within the P82 amplicon (V147), were analyzed in two A-M31 chromosomes (samples 5 and 6 in Table S1). This analysis led to the splitting of A1a into two branches that shared all but four of the 23 mutations analyzed (Figure 1).

#### Haplogroup A2

Haplogroup A2 was first reported as a clade limited to Khoisan-speaking populations in southern Africa [26]. Recently, a new deep A2 paragroup was found among western Pygmies from central Africa by Batini et al. [19]. In the tree by Karafet et al. [14], haplogroup A2 is defined by P3 and other 17 phylogenetically equivalent markers, with three mutations that define three terminal branches (A-M114, A-P28 and A-P262). Here, 19 mutations that had been identified for haplogroup A2 [16], as well as the markers P3, M114, P28, P262, were genotyped in three A2 Y chromosomes (samples 7–9 in Table S1). This analysis made it possible to identify a new branch (A-V218) within haplogroup A-P262. The newly reported V73 mutation was found to be phylogenetically equivalent to M114 (Figure 1).

#### Haplogroup A3

In the tree by Karafet et al. [14], haplogroup A3 is defined by the M32 mutation and contains two African-specific clades, A3a and A3b. A3a is a rare branch that has been observed exclusively in eastern Africa [19]–[21]. The sister clade, A3b, is further subdivided into a southern African (A3b1 or A-M51) and a mainly eastern African (A3b2 or A-M13) haplogroup [19]–[21], [23], [27]–[30]. Since both A3b1 and A3b2 are quite common, but are yet poorly resolved haplogroups, we performed a MSY re-sequencing analysis of about 90 kb for each of two A3b chromosomes (one A-M51* and one A-M13*) to find additional informative markers. We detected a total of 9 new mutations (V262–V317 in Table 1). A total of 41 markers (9 new mutations, 7 mutations from our database, 15 mutations identified in a previous study [16], and 10 mutations defining A3 branches in Karafet et al. [14]) were analyzed in ten subjects (samples 10–19 in Table S1). Mutation V249 joined haplogroups A2 and A3 and should be considered to be phylogenetically equivalent to the PK1 marker, which was originally thought to be only associated with the A2 lineage, but which has recently been found to cluster haplogroups A2 and A3 [19]. The phylogenetic mapping of the other mutations led to the identification of five new haplogroups, doubling the number of both A3b1 and A3b2 terminal branches. Finally, the P289 marker [14] was positioned upstream of both A3a and A3b (Figure 1).

#### Haplogroup B

Haplogroup B (B-M60 [14], [20]) is essentially a sub-Saharan clade. The majority of haplogroup B lineages show well defined and restricted geographic and ethnic distributions. An exception is represented by haplogroup B-M109, which is present in central, eastern, and southern Africa [19]–[21], [23], [27], [29], [30]. Extensive sequencing of one B-M109 chromosome recently led to the identification of 17 mutations for this haplogroup [16]. Here, a total of 33 mutations (17 previously described mutations [16], 2 mutations - V254 and V341 - identified during the mapping process, and 14 mutations defining haplogroup B branches in Karafet et al. [14]) were analyzed in 13 haplogroup B chromosomes (Table S1, samples 20–32). We substantially increased the resolution of the B2a clade (B-M150), with five new branches detected (Figure 1). The trifurcation B2a/B2b/B2c within the major clade B2 reported by Karafet et al. [14] was resolved by repositioning the M112 mutation (Figure 1).

#### “Out of Africa” haplogroups

All Y-clades that are not exclusively African belong to the macro-haplogroup CT, which is defined by mutations M168, M294 and P9.1 [14], [31] and is subdivided into two major clades, DE and CF [1], [14]. In a recent study [16], sequencing of two chromosomes belonging to haplogroups C and R, led to the identification of 25 new mutations, eleven of which were in the C-chromosome and seven in the R-chromosome. Here, the seven mutations which were found to be shared by chromosomes of haplogroups C and R [16], were also found to be present in one DE sample (sample 33 in Table S1), and positioned at the root of macro-haplogroup CT (Figure 1 and Figure S1). Six haplogroup C chromosomes (samples 34–39 in Table S1) were analyzed for the eleven haplogroup C-specific mutations [16] and for SNPs defining branches C1 to C6 in the tree by Karafet et al. [14] (Figure S1). Through this analysis we identified a chromosome from southern Europe as a new deep branch within haplogroup C (C-V20 or C7, Figure S1). Previously, only a few examples of C chromosomes (only defined by the marker RPS4Y_711_) had been found in southern Europe [32], [33]. To improve our knowledge regarding the distribution of haplogroup C in Europe, we surveyed 1965 European subjects for the mutation RPS4Y_711_ and identified one additional haplogroup C chromosome from southern Europe, which has also been classified as C7 (data not shown). Further studies are needed to establish whether C7 chromosomes are the relics of an ancient European gene pool or the signal of a recent geographical spread from Asia. Two mutations, V248 and V87, which had never been previously described, were found to be specific to haplogroups C2 and C3, respectively (Figure S1). Three of the seven R-specific mutations (V45, V69 and V88) were previously mapped within haplogroup R [34], whereas the remaining four mutations have been here positioned at the root of haplogroups F (V186 and V205), K (V104) and P (V231) (Figure S1) through the analysis of 12 haplogroup F samples (samples 40–51, in Table S1).

## Discussion

Here we report the mapping of 227 Y chromosome mutations mainly belonging to the African specific clades, greatly increasing the level of resolution for the deepest portion of the tree.

The structure of haplogroup A3b has been greatly refined. Based on the relative number of mutations accumulated, both the southern African A3b1 (A-M51) and the eastern African A3b2 (A-M13) clades seem to have experienced relatively recent expansions resulting in multiple terminal short sister clades. As for haplogroup B, a recent analysis of the microsatellite diversity of the B2a (B-M150) clade had shown a relatively deep coalescence for this haplogroup, suggesting a scenario that predates the diffusion of the Bantu languages [19]. Here, the fine dissection of the B2a clade, with five new branches identified, opens up the possibility to better understand the phylogeographic pattern associated with this widespread and still poorly studied sub-Saharan clade. Finally, the basal A1b haplogroup, an extremely rare haplogroup [16], [19], appears to be subdivided into a number of deep branches (Figure 1), a finding that highlights the importance of targeted studies of rare haplogroups in phylogenetic analyses. This haplogroup represents one of the two deepest-rooting branches of the most recently reported MSY tree [16]. New whole-chromosome African sequences are likely to provide a finer resolution of the basal portion of the Y chromosome phylogeny and, possibly, more deep-rooting Y chromosome branches.

Two A1b chromosomes from a previous work (one from Algeria and one from Cameroon) [16] were included in this study together with two newly identified A1b chromosomes, whose geographic origin can be traced back to west-central Africa (Ghana) on the basis of the microsatellite profile (data not shown). It is worth noting that three additional A1b chromosomes have been recently found in Caribbean populations, which exhibit substantial Y-STR haplotype sharing with Y chromosomes from Gabon [35], [36]. Taken together, all these data reinforce the hypothesis of an origin in the north-western quadrant of the African continent for the A1b haplogroup [16], and, together with recent findings of ancient Y-lineages in central-western Africa [19], provide new evidence regarding the geographical origin of human MSY diversity.

Different geographical regions in the African continent have been proposed for the origin of different portions of the human genome [18], [37], [38]. Current whole-genome sequencing projects [e.g. 15,39] and new methods to analyze ancient DNA samples [40] are likely to provide a more coherent picture of the demographic history and origin of our species.

## Materials and Methods

### Ethics Statement

This study is part of a research project approved by the “Policlinico Umberto I, Sapienza Università di Roma” Ethical Committee (protocol number 1016/10, according to the DM 15/7/1997 and following). The data were analyzed anonymously.

### DNA Samples

Samples were chosen on the basis of their SNP/microsatellite genotype as determined in the present or previous studies [21], [41]. A total of 51 DNA samples from the collections of the Authors were analyzed. Haplogroup information and country of origin of each sample is reported in Table S1. Haplogroup nomenclature follows the same criterion as reported in [16].

### Identification of New Mutations by Sequencing

Overall, about 90 Kb were sequenced for each of two unrelated Y chromosomes belonging to haplogroups A3b1 (89.8 kb) and A3b2 (89.3 kb).

We designed polymerase chain reaction (PCR) and sequence primers on the basis of the Y-chromosome sequence reported in the February 2009 assembly of the UCSC Genome Browser (http://genome.ucsc.edu/) using Primer3 software (http://frodo.wi.mit.edu/primer3/). Sequencing templates were obtained through PCR in a 50-µl reaction containing 50 ng of genomic DNA, 200 µM each deoxyribonucleotide (dNTP), 2.5 mM MgCl_2_, 1 unit of Taq polymerase, and 10 pmoles of each primer. A touch-down PCR program was used with an annealing temperature that decreased from 62°C to 55°C over 14 cycles, followed by 30 cycles with an annealing temperature of 55°C.

Following DNA amplification, PCR products were purified using theQIAquick PCR purification kit (Qiagen, Hilden, Germany). Cycle sequencing was performed using the BigDye Terminator Cycle Sequencing Kit with Amplitaq DNA polymerase (Applied Biosystems, Foster City, CA) and an internal or PCR primer. Cycle sequencing products were purified by ethanol precipitation and run on an ABI Prism 3730XL DNA sequencer (Applied Biosystems). Chromatograms were aligned and analyzed for mutations using Sequencher 4.8 (Gene Codes Corporation, Ann Arbor, MI).

### Genotyping and Phylogenetic Mapping

To obtain a refined MSY tree, we determined the allelic state at 168 markers (22 newly described mutations and 146 previously reported mutations [16]) in 51 Y chromosomes which were representative of different Y haplogroups (Table S1). Fifty-nine mutations reported by Karafet et al. [14] were also analyzed (Figure 1 and Figure S1). In order to detect the presence, if any, of the intermediate variant allele DYS449.2 [42], nineteen Y*(xBT) and 4 B chromosomes were typed for the DYS449 microsatellite by sequencing.

## Supporting Information

Figure S1 **Structure of the macro-haplogroup CT.** For details on mutations see legend to Figure 1. Dashed lines indicate putative branchings (no positive control available). The position of V248 (haplogroup C2) and V87 (haplogroup C3) compared to mutations that define internal branches was not determined. Note that mutations V45, V69 and V88 have been previously mapped (Cruciani et al. 2010; Eur J Hum Genet 18∶800–807).
(TIF)

Table S1 **Haplogroup affiliation for 51 Y chromosomes analyzed in this study.**
(XLS)
