# Supplementary figures and images for: Molecular Dissection of the Basal Clades in the Human Y Chromosome Phylogenetic Tree
Source: PLoS One. 2012 Nov 7;7(11):e49170. doi: 10.1371/journal.pone.0049170 (PMC3492319; doi:10.1371/journal.pone.0049170)

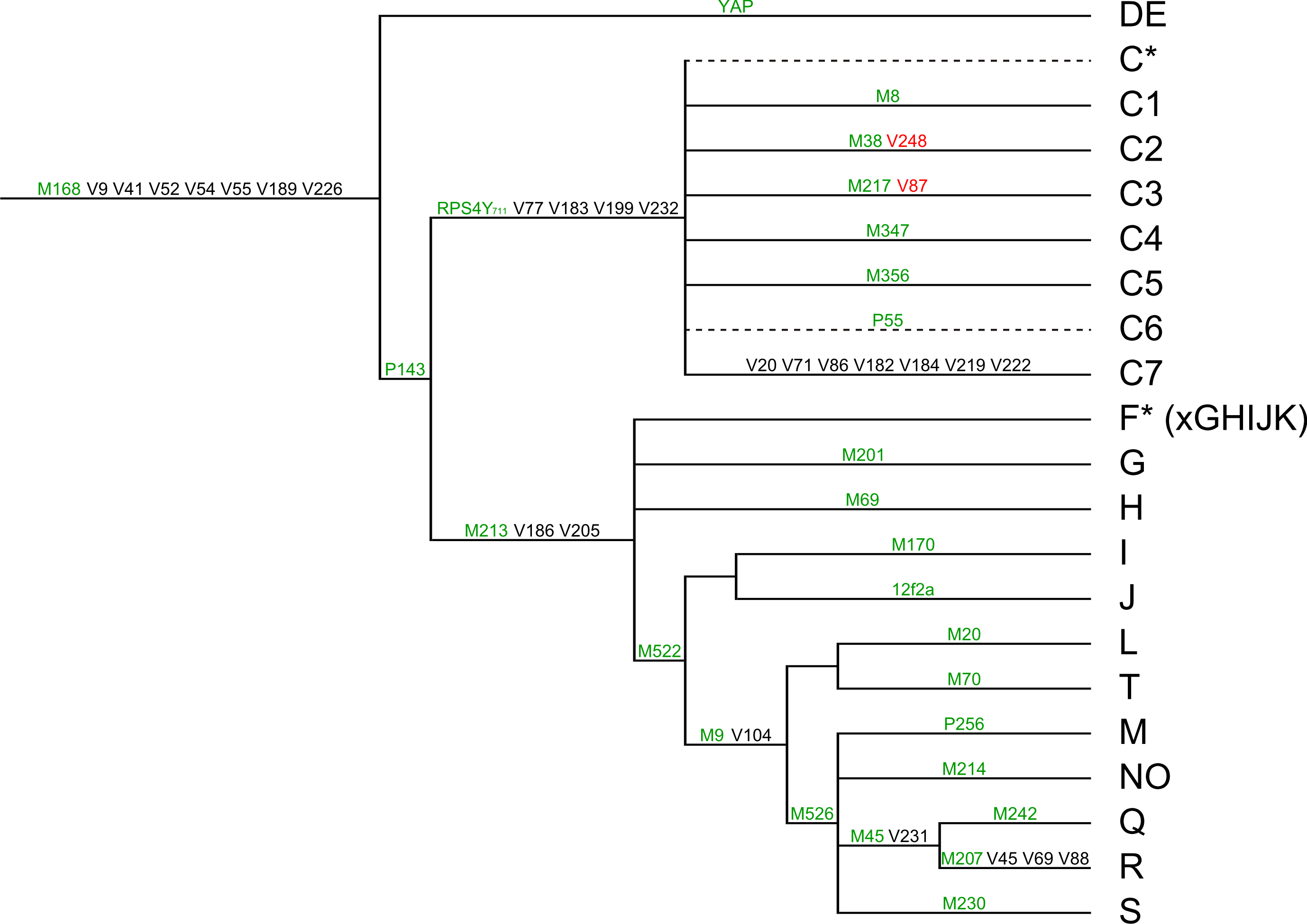

Supplement: Figure S1 — Structure of the macro-haplogroup CT. For details on mutations see legend to Figure 1. Dashed lines indicate putative branchings (no positive control available). The position of V248 (haplogroup C2) and V87 (haplogroup C3) compared to mutations that define internal branches was not determined. Note that mutations V45, V69 and V88 have been previously mapped (Cruciani et al. 2010; Eur J Hum Genet 18∶800–807). (TIF) [file pone.0049170.s001.tif]
